# Classifying literature mentions of biological pathogens as experimentally studied using natural language processing

**DOI:** 10.1186/s13326-023-00282-y

**Published:** 2023-01-31

**Authors:** Antonio Jose Jimeno Yepes, Karin Verspoor

**Affiliations:** 1 School of Computing Technologies, RMIT University, Melbourne, Australia; 2 School of Computing and Information Systems, The University of Melbourne, Melbourne, Australia

**Keywords:** Pathogen characterisation, Data set generation, Scientific literature, Natural language processing, Text mining

## Abstract

**Background:**

Information pertaining to mechanisms, management and treatment of disease-causing pathogens including viruses and bacteria is readily available from research publications indexed in MEDLINE. However, identifying the literature that specifically characterises these pathogens and their properties based on experimental research, important for understanding of the molecular basis of diseases caused by these agents, requires sifting through a large number of articles to exclude incidental mentions of the pathogens, or references to pathogens in other non-experimental contexts such as public health.

**Objective:**

In this work, we lay the foundations for the development of automatic methods for characterising mentions of pathogens in scientific literature, focusing on the task of identifying research that involves the experimental study of a pathogen in an experimental context. There are no manually annotated pathogen corpora available for this purpose, while such resources are necessary to support the development of machine learning-based models. We therefore aim to fill this gap, producing a large data set automatically from MEDLINE under some simplifying assumptions for the task definition, and using it to explore automatic methods that specifically support the detection of experimentally studied pathogen mentions in research publications.

**Methods:**

We developed a pathogen mention characterisation literature data set —READBiomed-Pathogens— automatically using NCBI resources, which we make available. Resources such as the NCBI Taxonomy, MeSH and GenBank can be used effectively to identify relevant literature about experimentally researched pathogens, more specifically using MeSH to link to MEDLINE citations including titles and abstracts with experimentally researched pathogens. We experiment with several machine learning-based natural language processing (NLP) algorithms leveraging this data set as training data, to model the task of detecting papers that specifically describe experimental study of a pathogen.

**Results:**

We show that our data set READBiomed-Pathogens can be used to explore natural language processing configurations for experimental pathogen mention characterisation. READBiomed-Pathogens includes citations related to organisms including bacteria, viruses, and a small number of toxins and other disease-causing agents.

**Conclusions:**

We studied the characterisation of experimentally studied pathogens in scientific literature, developing several natural language processing methods supported by an automatically developed data set. As a core contribution of the work, we presented a methodology to automatically construct a data set for pathogen identification using existing biomedical resources. The data set and the annotation code are made publicly available. Performance of the pathogen mention identification and characterisation algorithms were additionally evaluated on a small manually annotated data set shows that the data set that we have generated allows characterising pathogens of interest.

**Trial registration:**

N/A.

**Supplementary Information:**

The online version contains supplementary material available at 10.1186/s13326-023-00282-y.

## Introduction

Pathogens are organisms that can cause disease. Most typically, the term refers to microorganisms such as viruses and bacteria that are ubiquitous within our bodies (e.g. in the gut) and in our environment [1]. Most of these microorganisms are harmless, but some are pathogenic, i.e. causing disease. The interactions between humans and biological pathogens have shaped human history and affected the way we live, with COVID-19 being a recent example.

The study of pathogens has many practical applications, from identifying the disease mechanisms of a pathogen [2] to understanding antibiotic resistance [3]. Furthermore, pathogens are relevant in the development of new biomaterials [4, 5] and have applications in other non-pathogenic scenarios [6]. New discoveries related to pathogens are made available in the scientific literature, which is growing at a rapid pace with over 1 million articles added to MEDLINE every year. This is certainly the case with COVID-19 related literature, for which new research is quickly emerging in large volumes [7, 8].

The identification of scientific literature on pathogens, in particular research that involves experimental study of their molecular properties and disease mechanisms, can provide timely information for pathogen management and treatment of the diseases they cause. Furthermore, retrieval and analysis of literature describing such experimental use of pathogens can also support identifying who is doing research on specific pathogens in the context of biosurveillance [9].

Currently, retrieval of literature relevant to specific pathogens typically relies on keyword search of the pathogen names, and results in many spurious matches to articles where a pathogen may be mentioned although it is not a primary focus of the research presented, or not studied directly through experimental methods. In this work, we seek to lay the foundation for automatic methods for identifying literature relating to molecular study of pathogens. There are databases with curated biomolecular information about pathogens, with the scientific literature a primary source of this information. On the other hand, there is no explicit manually annotated data set available to train or evaluate natural language processing (NLP) methods that aim to extract information from the literature and transfer it to these databases.

In this article, we propose several strategies for utilising existing biomedical resources to build a data set that can be used to make decisions on the design of natural language processing methods for the identification of literature relevant to pathogen curation. Since there is no large ground-truth set readily available for the experimental pathogen mention characterisation task, we have automatically constructed a large data set from existing public NCBI resources. To facilitate this, we make the simplifying assumption that the indexing of an article citation with a MeSH term for the pathogen corresponds to the notion of an experimental pathogen in our target task. We make this READBioMed-Pathogens data set available at [10]. Additional information about the data sets and code is available in Additional file 1 Appendix A of the supplementary material.

Using our data set, we also present our approach for addressing this task of experimental pathogen mention characterisation. In pathogen mention characterisation, we aim to identify mentions of pathogens that are the subject of experimental, typically molecular, research. We wish to ignore mentions of pathogens that are mentioned in non-experimental contexts, i.e. for which there is no evidence that the researchers presenting the work held and studied molecular samples of the pathogen in their facility. Such non-experimental mentions may occur for several reasons in a paper, including for comparison with the primary pathogen under study, or in discussion of related work. These mentions can be considered ancillary to the primary contributions of the research.

The outcome of the pathogen mention characterisation is to select only the primary pathogens experimentally studied in a research paper. This can support indexing of the scientific literature for targeted retrieval of work directly relevant to a pathogen, or enable summarisation, which requires focusing only on the key contributions of a paper. For instance, all entities identified in the paper can be used to create an index [10] or entity-oriented retrieval strategies involving query expansion may be employed [11].

In our approach, we split the pathogen mention characterisation task into two steps (a) pathogen mention identification and (b) filtering of the pathogen mentions that are not directly related to understanding of the pathogen.

To address the limitations of our automatic approach to construction of the large data set, we also test the developed methods on a small manually annotated data set with pathogens annotated at the document level, showing the performance of various configurations of the proposed methods.

## Related work

Pathogen information has been curated into several existing databases. One key resource is the NCBI Taxonomy, which provides a reference set of biological organisms, and their taxonomic classification. We can identify publicly available resources specific to pathogen detection, e.g. NCBI Pathogen Detection [12], the study of pathogenic phenotyping, e.g. PathoPhenDB [2], and toxins related to pathogens, e.g. TADB2.0 [13], the bacteria type II toxin-antitoxin database. There are also other resources that are not publicly available [14], including the Biological Materials Information Program (BMIP).

The scientific literature contains information about research on pathogens and the research institutes performing research on them through, e.g. author affiliations. There is previous work in identifying pathogens in the scientific literature, which tends to focus on specific pathogens and/or specific aspects of pathogens. Among this work we can point to the Bacteria Biotope challenge task at the BioNLP shared tasks [15], which focuses on certain bacteria and their habitats and phenotypes, including 491 individual microorganisms mentioned in 392 articles. There is previous work using the literature to identify the relation of pathogens to the environment [16], pathogen-disease prediction using ontologies and literature mining [17], identification of the geolocation of pathogen samples (e.g. GeoBoost [18, 19]) for phylogeography or other aspects of pathogens related to biodiversity [20, 21], in addition to toxins [13, 22].

Despite this existing work for the identification of pathogen mentions in the scientific literature, there is no comprehensive work on characterising a large set of different pathogen types, or focusing on literature describing the experimental study of pathogens that can be used to evaluate pathogen annotation methods or to annotate a broad set of microorganisms, including pathogenic organisms, PrPSc prions and toxins.

In terms of broad objective as well as methodologically, our work is related to the Chemical Indexing task of the recent BioCreative NLM-Chem track [23]. To construct the NLM-Chem dataset [24], Medical Subject Heading (MeSH) index terms corresponding to chemicals are assigned to an article as a topic term. Similarly, we leverage MeSH index terms as a proxy for identifying key entities in articles in our automatically constructed dataset. The computational task in each case is primarily to identify the topic/key entities mentioned an article. In our work, we focus on biological pathogens rather than chemicals, and aim for a narrower definition of the entities that we consider relevant. Our dataset also takes advantage of resources beyond MeSH index terms over PubMed, to identify literature for a substantially broader set of pathogens.

In the following sections, we present a methodology to develop a data set that can be used to tune and evaluate pathogen identification methods. Then, we evaluate several methods to identify and characterise experimentally studied pathogens that include dictionary methods and state-of-the-art deep learning methods, based on our constructed data set.

### Methods

In this section, we describe the methodology used to construct the READBiomed-Pathogens pathogen literature data set that we use to develop and evaluate methods for pathogen identification and characterisation. This data set consists of MEDLINE/PubMed citation records; the texts that we analyse are the title and abstract texts within these records.

Within the set of microscopic organisms, our work considers specific types of pathogens that are classified within the NCBI Taxonomy [25]. The most relevant organism types are bacteria, fungi, protozoa, viroids and viruses [26, 27]. We have recovered information about a set of common pathogenic organisms and selection of less frequent ones that were found in the NCBI Taxonomy at the species level.

We have also considered other pathogens that cannot be categorised within an organism taxonomy but are still relevant to be studied, such as PrPSc prions [28], which are misfolded proteins that cause diseases such as Creutzfeldt-Jakob disease. We have considered prions of common species. As well, we have considered a set of common toxins generated by other pathogens including bacteria or fungi, such as enterotoxins that are produced and secreted by bacteria [29].

Finally, the pathogens represented in the READBiomed-Pathogens data set can be split into three main categories: pathogenic organisms (2848 terms), PrPSc prions (14 terms) and toxins (19 terms).

### READBiomed-pathogens data set generation

In this section, we describe how we generated READBiomed-Pathogens, leveraging existing resources from the National Center for Biotechnology Information (NCBI), which is part of the US NIH / National Library of Medicine, using the E-utilities [30].

To develop this data set, we are specifically interested in recovering literature citations that are relevant to the pathogens of interest. We draw on other NCBI resources, including Medical Subject Headings (MeSH) [31] corresponding to pathogen terms. MeSH headings are assigned manually to MEDLINE citations and provide highly reliable labels reflecting key topics addressed in publications. More recent MEDLINE records combine manual annotation with automatic annotation, but our data set was developed before this automatic MeSH indexing was put in place. This might need to be considered by future work following our approach.

**Fig. 1:**
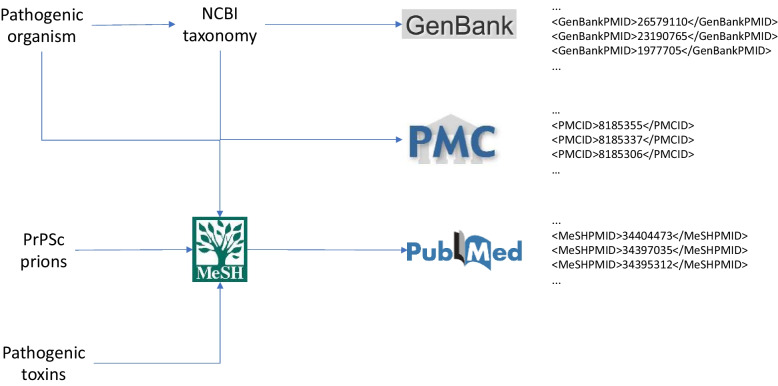
Diagram of the generation of the READBiomed-pathogens data set

Figure 1 shows a diagram of the process that we followed to create our data set, which is further explained in the following sections. We considered three types of pathogens – a pathogenic organism, prion proteins that cause infectious disease, and pathogenic toxins.

The NCBI offers other relevant resources to identify additional relevant scientific articles. In the case of the pathogenic organisms, GenBank [32] is a gene database with links to PubMed and allows recovering citations in which genes related to the pathogenic organisms have been identified in the scientific literature. Depending on the pathogen type, we queried different data sources to obtain document identifiers from PubMed or PubMed Central. We used these identifiers to build a data set with relevant literature for the pathogens of interest. This methodology can be straightforwardly applied for additional pathogens not considered in the current study.

In constructing our dataset, we have adopted two simplifying assumptions about the relationship between experimentally studied pathogens and the literature:

If a pathogen is included as a MeSH index term for an article in PubMed, then it is a focus entity of the research described in that article, and it is experimentally studied.

If a GenBank record for a pathogen links to an article in PubMed, then the pathogen is a focus entity of the research described in that article, and it is experimentally studied.

While it is clear that these assumptions are overly simplistic, they provide a reasonable proxy for our target task that allows us to conduct larger-scale computational experiments with machine learning methods.

### Pathogenic organisms

We grouped pathogens corresponding to biological organisms, including bacteria, fungi, protozoa, viroids, and viruses, together, since most species are directly available from the NCBI Taxonomy [33]. The NCBI Taxonomy contains most of the pathogenic organisms of interest. To find the pathogenic organisms in the NCBI taxonomy database, we searched for the name of a pathogen in the NCBI taxonomy vocabulary, first seeking to match a scientific name. If there was no match, then we expanded the search into all name fields available in the NCBI Taxonomy database. We only consider cases in which a single NCBI Taxonomy record was returned.

For each NCBI Taxonomy pathogen recovered, we obtained pathogen synonyms, a list of all strains in the NCBI Taxonomy database and identifiers of the pathogen from other resources provided by NCBI such as the MeSH controlled vocabulary and GenBank. To obtain article identifiers (PubMed or PubMed Central IDs), we searched PubMed for citations indexed with MeSH terms corresponding to the pathogen identifiers recovered from the NCBI taxonomy record and extracted direct mappings to PubMed from GenBank records linked to from the NCBI Taxonomy.

As already mentioned, for each pathogen identified in the NCBI Taxonomy database, we recovered all the subspecies identifiers using recursive queries. Information recovered for the subspecies was added to the pathogen record to encompass all possible variants of each pathogen as fully as feasible.

### PrPSc prions data set

Prions are misfolded proteins that produce diseases such as Creutzfeldt-Jakob disease. We are interested in pathogenic prions such as the scrapie isoform of proteins (PrPSc) associated to specific animal species such as sheep, human or moose. To recover citations relevant to PrPSc and the species of interest, we identified MeSH indexing as a key resource. While there is no entry in MeSH for variants of PrPSc prions, a MeSH heading for PrPSc prions in general, as well as for each of the species, is available. Since specific prion types do not appear as entries in MeSH, in order to recover MEDLINE citations relevant to PrPSc proteins for humans, we utilize a template query {“PrPSc Proteins”[MH] AND humans [MH]}.

Of the 14 prions of interest, it was possible to collect documents for 7 of them. The following species were not found in MeSH: elk, greater kudu, moose, mule, nyala, onyx and ostrich. To collect relevant citations for the data set, we reused the query example presented above as a template. We collected the citations in MEDLINE that were identified by this template for each one of the species.

### Toxins data set

Toxins, even if some are related to pathogens, are chemicals and hence do not appear in the NCBI Taxonomy database. Therefore, we explored the MeSH controlled vocabulary as a resource for toxins indexing in MEDLINE.

13 out of the 19 toxins in our list of pathogens were not found in MeSH: Abrus abrin toxin, Anatoxin-A, Batrachotoxin, Brevetoxin, decarbamoylsaxitoxin, Fusariotoxins (T-2), gonyautoxins, Maitotoxin, Mycotoxin, neosaxitoxin, Palytoxin, and Ricinus ricin toxin. For the 6 toxins that could be mapped to a MeSH entry, we added the citations that were indexed with that toxin in PubMed to our data set.

Additional work could extend our set of toxins to the ones available in chemical databases such as ChEBI (Chemical Entities of Biological Interest).

### READBiomed-pathogens data set statistics

In this section, we provide statistics of the data collected for the different categories of pathogens in Table 1. It was not possible to find PubMed citations for 122 pathogenic organisms in the NCBI Taxonomy database, which in most cases are viruses, e.g. *viper retrovirus*. We found that just over 10% of all pathogenic organisms were available as MeSH headings, in comparison to the pathogens available in GenBank. On the other hand, the number of citations available per pathogen is larger in MeSH. Despite these differences in the information contained in each database, an advantage of using MEDLINE MeSH indexing is that articles have been manually indexed. Hence, we can determine the articles in which the pathogen has been identified as sufficiently relevant to be included in the index terms. This allows us to identify which MEDLINE citations we should consider when evaluating pathogen characterisation algorithms.

**Table 1:** Statistics of READBiomed-Pathogens. This includes the number of pathogens identified in the resources MeSH and GenBank compared to the total number of pathogens in our set of interest (Total)), and the average number of PubMed citations (Avg. PMIDs) associated to each pathogen in MeSH and GenBank

Group	Total	MeSH	Avg. PMIDs	GenBank	Avg. PMIDs
Organism	2679	360	3901.26	1785	242.36
PrPsc	14	7	346	–	–
Toxins	19	6	11,730	–	–

Table 2 shows the top pathogenic organisms sorted by the number of unique citations recovered from MeSH indexing or GenBank. The bacteria *Escherichia coli* is the pathogen with the most citations from both sources. Most of these frequent pathogens appear in MeSH but the ranking is different in the two lists, reflecting differences in scope.

**Table 2:** Top 10 pathogenic organisms identified from MeSH indexing and GenBank sorted by number of PubMed identifiers recovered from the NBCI resources. (*) *Salmonella enterica* subsp. enterica serovar Typhimurium

MeSH indexing			GenBank		
NCBI id	Name	PMIDs	NCBI id	Name	PMIDs
562	*Escherichia coli*	288,697	562	*Escherichia coli*	39,836
1280	*Staphylococcus aureus*	80,265	11,676	Human immunodeficiency virus 1	3904
1773	*Mycobacterium tuberculosis*	52,603	1773	*Mycobacterium tuberculosis*	3822
28,901	*Salmonella enterica*	46,122	1423	*Bacillus subtilis*	3342
11,320	Influenza A virus	45,982	1280	*Staphylococcus aureus*	2731
287	*Pseudomonas aeruginosa*	44,739	287	*Pseudomonas aeruginosa*	2413
210	*Helicobacter pylori*	35,576	624	*Shigella sonnei*	2240
5833	Plasmodium falciparum	30,357	294	*Pseudomonas fluorescens*	2236
90,371	*Salmonella enterica* subsp. (*)	28,902	621	*Shigella boydii*	2207
10,407	Hepatitis B virus	27,946	622	*Shigella dysenteriae*	2190

### Pathogen characterisation

In our work, we define experimental pathogen characterisation as the identification of a pathogen in the text that is experimentally studied in the published work. That is, we aim to ignore pathogens that are mentioned, but for which there is no evidence that the researchers presenting the work had actively experimented on the pathogen in their research, and hence held samples in their facility. Irrelevant pathogen mentions may occur in the context of references to previous or similar work, e.g. in background information, or they may be mentioned in the context of a comparison between organisms. For example, in the citation PMID:13129609, *Escherichia Coli* is mentioned repeatedly in the article, but the experimentally studied pathogen is *Proteus mirabilis*. *E. coli* supports the research on *Proteus mirabilis*. As another example, PMID:21979562 mentions H1N1 but it is in the context of the patient presentation. The patient had received a vaccine against the H1N1 virus. Additional examples are available in Additional file 1 Appendix C in the supplementary material.

The NLP methods were developed using the UIMA (Unstructured Information Management Application) framework [34]. Pathogen characterisation was split into two steps.

In the first step (pathogen identification), a specific case of named entity recognition of biological concepts [35], mentions of pathogens are identified in the text of the citations (title and abstract texts). Here, we utilise a dictionary method or regular expressions since the pathogen names are specific and derived from a closed vocabulary. The objective of this step is to identify as many mentions of pathogens in the texts as possible.

In the second step (pathogen filtering), the aim is to remove the pathogen mentions that are not relevant to the objective of identifying research that describes active experimentation with pathogens, and, conversely, to retain pathogen mentions that correspond to active experimentation.

### Pathogen identification

**Fig. 2:**
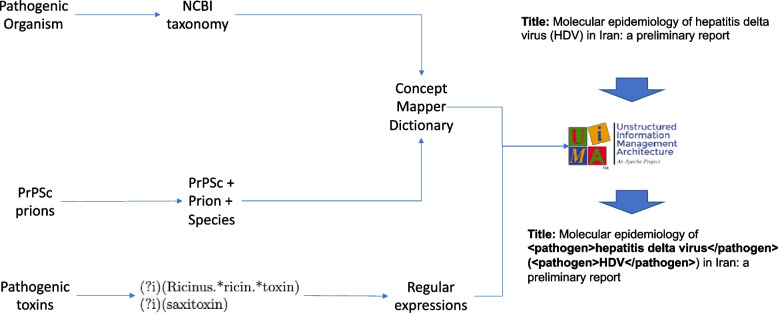
pathogen identification diagram. An input text is processed by a set of dictionary and regular expressions build on the pathogen list. UIMA is the NLP framework that we have used for the development of our method

We developed a methodology for the identification of pathogens in text. We followed distinct strategies depending on the pathogen type, as shown in Fig. 2. We used UIMA [36] as the framework to develop the pathogen identification components. We explain it in more detail in this section.

For pathogenic organism identification, we built a dictionary matching approach using a tool called ConceptMapper [36], which is part of the UIMA sandbox tools.^1^ ConceptMapper is highly configurable and scales well with large dictionaries. It has previously been shown to be highly effective for recognising biological concepts in scientific publications [37]. We evaluated several of the ConceptMapper options, together with a dictionary containing pathogenic organism terms and relevant terms for the identification of PrPSc prions.

For the generation of the dictionary for pathogenic organisms, we used a version of the NCBI Taxonomy available from the OBO Foundry. For each pathogenic organism, we selected the terms for the pathogen of interest and all its subspecies.

We made some modifications to the extracted terms from the OBO NCBI Taxonomy extracted terms. To increase recall, the word “subtype” was removed, e.g., “H1N1 subtype” becomes “H1N1”. Additionally, to reduce the size of the dictionary, we removed terms starting with “influenza a virus (”, “influenza b virus (” or “influenza b virus (” which removed several thousand entries with no impact on recall. For viruses, we removed the ending “virus”, which reduced the recall since in most cases the virus is implied, e.g. “influenza A” vs. “influenza A virus”. Additionally, we removed virus names with one letter, e.g. “B virus” from “Hepatitis B virus” which could annotate “Influenza B virus” incorrectly. After this processing, only terms with more than three letters were kept. Abbreviations are ambiguous and results showed that most of the abbreviations had long forms that matched one of the dictionary entries for that pathogen in the MEDLINE citations.

We have 2637 pathogenic organisms in our dictionary and a total of 83,757 distinct terms, with an average of 31 terms per pathogen. A large number of terms is justified as well by the term variation contributed by the subspecies, e.g. “*Borrelia burgdorferi* strain N40” for “*Borrelia burgdorferi*”. Since the longest match is preferred, the term linked to subspecies will be preferred.

For this PrPSc, we have split the set of PrPSc prions into a tuning set that includes the pathogens Sc (cattle), Sc (cat), Sc (deer) and Sc (goat). The testing set includes the pathogens Sc (human), Sc (mink) and Sc (sheep). Table 3 shows the results on the selected tuning set. We identify that missed PrPSc annotations are mostly due to missing species mentions in the citations. This is especially a problem in citations with no abstract. There are also wrong annotations, according to MeSH indexing, where another species might be mentioned in the citation that is not relevant to the research but is mentioned as background information.

**Table 3:** PrPSc identification algorithm results for tuning data set

Term	TP	Positives	FP + TP	Precision	Recall	F1
Sc (cat)	4	9	6	0.6667	0.4444	0.5333
Sc (cattle)	145	457	170	0.8529	0.3173	0.4625
Sc (deer)	29	46	40	0.7250	0.6304	0.6744
Sc (goat)	31	68	63	0.4921	0.4559	0.4733

For toxin identification, we initially generated a dictionary using the name of the toxin and additional terms using their mapping to the MeSH controlled vocabulary, when available. As in the case of the PrPSc prions, we have split the toxins that could be mapped to MeSH into tuning and testing. The training toxins are: aflatoxins, botulinum toxins, ciguatoxins and conotoxins. The testing toxins are: enterotoxins, saxitoxins and tetrodotoxin. Table 4 shows the results of applying the dictionary to the tuning toxins. We find that the precision tends to be quite high, which might indicate that if a toxin appears in the citation, it is very likely to be relevant. We find as well that some mentions are missed due to term variability. For instance, the pathogen aflatoxins might appear in text as “antiaflatoxinB1”. Another example is botulinum toxin, that might appear in text as “onabotulinumtoxinA” or “botulinum neurotoxin E”. We could expand the dictionary, but a change in the matching of the terms should be made to allow for a more flexible matching, since in some cases, toxin names get combined with other terms or more specific terms are used. This approach has been used in prior work on gene name normalization [38].

**Table 4:** Toxin identification on tuning data set

**Dictionary**	**TP**	**Positives**	**FP + TP**	**Precision**	**Recall**	**F1**
aflatoxins	9106	10,131	9109	0.9997	0.8988	0.9466
botulinum	9903	16,780	9915	0.9988	0.5902	0.7419
ciguatoxins	386	529	390	0.9897	0.7297	0.8400
conotoxins	2340	3144	2453	0.9539	0.7443	0.8362
**Regex**	**TP**	**Positives**	**FP + TP**	**Precision**	**Recall**	**F1**
aflatoxins	9127	10,131	9130	0.9988	0.9009	0.9477
botulinum	13,664	16,780	13,697	0.9976	0.8143	0.8967
ciguatoxins	386	529	390	0.9897	0.7297	0.8400
conotoxins	2352	3144	2465	0.9542	0.7481	0.8387

We also approached matching toxin names using regular expressions. We implemented a set of regular expressions based on the names of the toxins, the regular expressions can be found in Additional file 1 Appendix B of the supplementary material. Toxin synonyms extracted from the MeSH controlled vocabulary have been added and the expressions match both uppercase and lowercase letters by adding the case insensitive match string (?i) within the regular expression. Results using the regular expressions appear in Table 4. Precision is the same while recall has increased significantly.

### Pathogen filtering

Pathogen identification methods described in the previous section find all possible mentions of pathogens in the MEDLINE citations. But not all the identified pathogens are described as experimentally studied in those citations. Some pathogens might be mentioned as part of the background of existing work and others might be mentioned but the researchers were not directly working with the pathogen (e.g., mentioned in the context of a review paper or research describing surveillance of a pathogen-related disease). We evaluate a strategy to identify relevant training data and examine several models. As stated above, we assume that if a pathogen appears as a MeSH heading in the indexing of the citation, it might be experimentally studied.

#### Data set for pathogen focus detection

We developed a data set that we used to identify mentions of pathogens mentioned in the citations as relevant or not to the research described in the citations, as determined by the MeSH terms assigned to a citation. These terms capture the key focus topics of the paper; inclusion of a pathogen name in the MeSH terms for a citation indicates that the pathogen is relevant to the core research contribution of the paper. This was created by identifying mentions of pathogens using the dictionary method described above, like the processing done in [39], and then deciding on their relevance based on the inclusion of the pathogen name in the set of MeSH index terms for the article. The mentions of the pathogens of interest were substituted within the text of the citation by a common text representation @PATHOGEN$. This resulted in a total of 133,076 examples.

#### Pathogen filtering methods

We used the data set developed in the previous section to train several classifiers based on supervised machine learning algorithms. We trained a linear Support Vector Machine (SVM) [40] using a stochastic gradient descent implementation using the modified Huber loss [41] suited for imbalanced data [42] and AdaBoostM1 [43] (using the MTIMLExtension package [44]).

For both methods, the text of the citations, which includes the title and the abstract, was tokenized, stop words were removed and both unigrams or unigrams and bigrams were used as features. This processing was done using the BinaryPipeFeatureExtractor from the MTIMLExtension package.

In addition, we fine-tuned a BERT [45] based classifier using HuggingFace pre-trained models [46]. We specifically used a pre-trained model named BioBERT [47], which has been pre-trained using biomedical literature. BERT has a limit of 512 tokens, so documents larger than 512 tokens were truncated. BERT-like models tokenize text using a Wordpiece algorithm that breaks words into several subwords.

Table 5 shows the performance of the machine learning algorithms on this set. We find that BERT has the best performance. These trained algorithms will be evaluated on the pathogen filtering results using the manually annotated set described in the Results section. The fine-tuned BioBERT models perform better than SVM and AdaBoostM1, the difference in performance is similar to other related MEDLINE categorization tasks [48, 49].

**Table 5:** Classification results

READBiomed-Pathogens	Precision	Recall	F1
SVM	0.8975	0.9450	0.9206
AdaBoostM1	0.8654	0.9682	0.9139
BERT classifier	0.9359	0.9631	0.9493

We experimented with enriching the text with additional information provided by the Scientific Discourse Tagger [50], based on evidence that discourse information may be valuable for our task [51]. Using these features with the learning algorithms did not provide a significant change in results and additional work is needed to identify the best way to leverage the discourse annotations.

## Results

In this section, we present the results obtained using the pathogen identification and characterisation methods on the data sets that we have developed automatically on a manually annotated data set.

### READBiomed-pathogens data set

Pathogen identification results for the pathogenic organisms for which we could recover the MeSH indexing and are available in Table 6. The dictionary described in the Methods section has been used to obtain these results. F1 results for macro and micro averaging (averaging by pathogen class or an overall average across all instances, respectively) are quite similar, while the largest differences are in the precision and recall values.

**Table 6:** Identification performance of dictionary-based pathogen identification on the READBiomed-Pathogens data set, considering more/less frequent pathogenic organisms separately. *More frequent* means the top 10 pathogens as shown in Table 2, while the remaining pathogens have been grouped in the *Less frequent* category

More frequent	Precision	Recall	F1
Macro-average	0.9198	0.7335	0.8161
Micro-average	0.9141	0.7209	0.8061
Less frequent	Precision	Recall	F1
Macro-average	0.7771	0.8063	0.7914
Micro-average	0.8790	0.7245	0.7943

Table 7 shows the results on the testing set of the PrPSc prion pathogens. We identify that missed annotations are mostly due to missing species mentions in the citations, which is specially a problem in citations with no abstract. There are also wrong predictions according to MeSH indexing, where another species might be mentioned in the citation that is not relevant to the research but is mentioned as background information.

**Table 7:** PrPSc identification algorithm results for testing data set

Term	TP	Positives	FP + TP	Precision	Recall	F1
Sc (human)	531	1266	604	0.8791	0.4194	0.5679
Sc (mink)	11	16	18	0.6111	0.6875	0.6471
Sc (sheep)	323	559	381	0.8478	0.5778	0.6872

Results for toxin identification are available from Table 8. Regular expressions have comparable precision to the dictionary approach, but the recall has slightly increased.

**Table 8:** Toxin identification on testing set

**Term**	**TP**	**Positives**	**FP + TP**	**Precision**	**Recall**	**F1**
enterotoxins	8477	25,679	8490	0.9985	0.3301	0.4962
saxitoxins	909	1270	980	0.9279	0.7157	0.8080
tetrodotoxins	7944	12,848	8109	0.9797	0.6183	0.7581
**Regex**	**TP**	**Positives**	**FP + TP**	**Precision**	**Recall**	**F1**
enterotoxins	8504	25,679	8517	0.9985	0.3312	0.4974
saxitoxins	939	1270	1012	0.9279	0.7394	0.8230
tetrodotoxins	7958	12,848	8123	0.9797	0.6194	0.7590

### Results on a small manually annotated set

We report results on a small additional data set of 87 manually annotated citations (again including title and abstract text only); these were annotated by pathogen biology experts in line with our objective to identify experimentally researched pathogens. Pathogenic organisms were assigned identifiers obtained from the NCBI Taxonomy database, and PrPSc prions and toxins have been assigned specific identifiers using the prefixes prpsc- or toxin- accordingly. Only two pathogens annotated in this corpus could not be mapped to the NCBI Taxonomy because they are very generic organisms (parainfluenza and adenovirus).

#### Pathogen identification

For pathogen identification, we used the best configuration based on the automatically generated set as presented in the previous section. Combining the outcome of the three methods, we obtain a precision of 0.5506, a recall of 0.8305 and a F1 of 0.6622, as shown in Table 9. The missing terms using the dictionary approach are due to term variation. For example, the term Influenza A and B viruses in citation PMID:25179390 refers to two virus types in a coordination. With the current dictionary approach, influenza virus A would be identified, while influenza B virus would be missed. Existing work [52] could be explored to identify more complex term variations.

**Table 9:** Pathogen characterisation results. Pathogen identification (PI) is filtered with either a SVM or a BERT classifier that identifies and removes non-relevant pathogen mentions (PF, pathogen focus)

Method	Precision	Recall	F1
Pathogen identification	0.5632	0.8305	0.6712
PI + PF filtering SVM	0.5581	0.8136	0.6621
PI + PF filtering BERT	0.6104	0.7966	0.6912

#### Pathogen characterisation

In the pathogen identification step, we aim to find as many pathogen mentions as possible in the citation texts, even though not all the correctly identified pathogens will be of interest for our broader objective of finding literature related to experimental study of those pathogens. Thus, we propose to filter irrelevant pathogen mentions after the identification step with a data set described in the Methods section. This data set was automatically generated leveraging MeSH indexing to identify pathogens that are not the main objective of the research mentioned in the citation. The filtering was adjusted based on prediction confidence of the classifier, which should be better tuned if a larger data set is made available.

Table 9 shows the result of the various evaluated methods. We see that the pathogen identification (PI) method has the largest recall, which is expected since the other methods are intended to increase precision (which might come at the cost of reduced recall). Using the pathogen focus (PF) filtering methods, the precision can be improved, which increases the F1 value with different recall reduction.

## Discussion

We have evaluated the identification of pathogens and their characterisation using two data sets, one generated by manual annotation and another one using existing biomedical resources. The READBiomed-Pathogens automatically generated data set is based on manually curated pathogen knowledge. Using this data set, we developed the dictionary and used the data to train machine learning algorithms to filter some of the dictionary annotations. Then, we tested the developed system on a manually annotated set, from where we evaluated the dictionary and trained models. The data set contains just a fraction of the pathogens of interest, which is due to the coverage of the resources used. MeSH is the only resource we could use to recover all types of pathogens of interest while GenBank was relevant to find relevant citations for pathogenic organisms. Even if all pathogens of interest were not identified in these sources, we could still use the available information to develop and tune our pathogen identification and characterisation algorithms.

We observe that the recall obtained evaluating our algorithm on the automatic data set citations is high, but there are still pathogens that were not identified. After manual analysis of randomly selected missed pathogen mentions we found that some of these pathogens were not mentioned in the citation. In some cases, citations contained a title but did not contain the abstract. Table 10 shows that approximately 19% of the citations have no abstract, or 10% if we consider the cases in which a pathogen is not found and there is no abstract. This justifies some of the missing pathogen mentions, which might be in the full text of the article.

**Table 10:** Distribution of pathogenic organism mentions. False negatives estimated using citations with missing pathogens after the dictionary method

Type	Count	Percentage
All documents	1,224,707	100.00%
With Open Access	125,265	10.23%
No Abstract	240,330	19.62%
With Abstract	984,377	80.38%
With Abstract and With Open Access	121,223	9.90%
No Abstract and With Open Access	4042	0.33%
With False Negatives	322,638	26.34%
With False Negatives and No Abstract	133,657	10.91%
With False Negatives and With Open Access	21,233	1.73%

MeSH indexing of MEDLINE is done by (primarily human) indexers that have access to the full text version of the article. This means that just using the citation information for pathogen characterisation might miss many mentions. On the other hand, full text articles are not as readily available as MEDLINE citations – only a small set of full text articles are available under an Open Access Creative Commons license provided by PMC. We have estimated the information that might be available as full text articles for pathogen characterisation. As shown in Table 10, from the total number of citations that we recovered using NCBI resources, we find that only 10% align with Open Access articles from PMC. From the set of citations where we identify false negatives, the overlap with PMC Open Access is less than 2%, which means that we could still identify a subset of full text articles we use for pathogen identification, but it will be a small fraction of what would be available from MEDLINE. From the citations, somewhat less than 20% have no abstract, so the pathogen characterisation relies solely on the title of the article. Unfortunately, the overlap of these articles with PMC Open Access is 0.33%, so there is no significant benefit to using the available full text articles.

We used the PMIDs recovered from MeSH indexing because we could obtain a set of positive and negative examples for pathogen identification and characterisation. From the information recovered from the NCBI resources, we did not use the PMIDs recovered from GenBank or PubMed Central. We observed that the PMIDs obtained from PubMed Central denote pathogens that appear in the full text, but do not necessarily need to be the focus of the paper. In the case of GenBank, it might be interesting to further explore the information from the referenced PMIDs.

For the pathogen identification task, we have considered the NCBI taxonomy from the OBO repository and the MeSH controlled vocabulary. On the other hand, there are additional resources in the OBO Foundry that could be used to extend the terms included in our dictionary. For instance, the NCI Thesaurus available from OBO could contribute with additional synonyms to some of the selected toxins, such as botulinum neurotoxin serotype E. We leave this for future expansion of the work.

We have evaluated an additional filtering method for pathogen characterisation. Articles might mention pathogens that are the focus of the article while the researchers that published the paper might have not directly worked experimentally with the pathogen, e.g., in review papers or for disease surveillance/prevalence analysis. There are several strategies that could potentially be used to identify these articles automatically, e.g., identifying if an article is a systematic review using MeSH indexing. However, there are many varied reasons for the mention of a pathogen not to be relevant for pathogen characterisation so this alone would be too narrow.

We therefore collected a set of random citations from the PMIDs recovered from NCBI resources and annotated them manually. From the 1000 retrieved citations, we removed (a) one that was incorrectly formatted, (b) citations that contained no abstract (e.g., PMID: 26268688 with title “An opportunity for further control of hepatitis B in China?” since the title does not provide enough context) – and (c) citations for which we could not determine the class label from the abstract text (e.g., PMID:16716232 with title “Topology and weights in a protein domain interaction network–a novel way to predict protein interactions”), which might also indicate that not all the information is available from the citation text.

After manual review and annotation, this set of non-relevant documents (NRDs) contained 793 citations, of which 502 were positive (relevant) and 281 were negative. We split this set into 523 training citations and 263 testing citations.

In Table 11, when combining the pathogen identification method and filtering using both models trained using the NRDs and the non-relevant pathogens / pathogen focus (PF) sets, we see the largest increase in precision and F1. These results show that it is possible to reuse available biomedical resources to prepare annotators prior to using task specific data.

**Table 11:** NRDs (non-relevant documents) and the non-relevant pathogens / pathogen focus (PF) sets

Manual set	Precision	Recall	F1
SVM	0.9022	0.9222	0.9121
AdaBoostM1	0.8333	0.9167	0.8730
BERT classifier	0.9158	0.9667	0.9405

There are several directions for extending the current work for pathogen characterisation (Table 12). In pathogen identification, it would be interesting to evaluate different methods to deal with term variability for pathogenic organisms. The data sets that we have automatically generated can help identify the variations with the largest gain in recall. In pathogen characterisation, further annotation of relevant citations with pathogens of interest would be of interest. The current manually annotated data set contains 87 citations, which are mostly focused on influenza.

**Table 12:** Pathogen characterisation results. Pathogen identification (PI) is filtered with either a SVM or a BERT classifier that identifies non-relevant documents (NRDs) or non-relevant pathogens / pathogen focus (PF)

Method	Precision	Recall	F1
Pathogen identification	0.5632	0.8305	0.6712
PI + NRD filtering SVM	0.6184	0.7966	0.6962
PI + NRD filtering BERT	0.6104	0.7966	0.6912
PI + PF filtering SVM	0.5581	0.8136	0.6621
PI + PF filtering BERT	0.6104	0.7966	0.6912
PI + NRDs SVM + PF BERT	0.6716	0.7627	0.7143

## Conclusions and future work

We studied the annotation of pathogens in scientific literature, developing several natural language processing methods supported by an automatically developed data set. As a core contribution of the work, we presented a methodology to automatically construct a data set for pathogen identification using existing biomedical resources, including NCBI databases and MeSH indexing of PubMed. The resulting data set, READBiomed-Pathogens, was used to develop and tune algorithms for pathogen identification and characterisation. The data set and the annotation code are publicly available.

Performance of the pathogen identification and characterisation algorithms were additionally evaluated on a small manually annotated data set shows that the data set that we have generated allows characterising pathogens of interest. Even though our work has focused on pathogenic microorganisms, the same methodology could in principle be used for other types of microorganisms not covered in our current list of pathogens.

MEDLINE citation data offers good coverage and a reasonable proxy for the pathogen characterisation task, based on the results obtained on the manually annotated data set. This is an important finding, since citation metadata is more widely available as compared to full text articles. The current work could also be extended to full text to estimate the coverage more precisely to allow for comparison of the information present in full text articles cf. their citations. Meaningful evaluation of this would require extending the manually annotated data set to full text articles. In addition, during the development of our data set, were covered and made publicly available information in relation to GenBank and PubMed Central, which can be considered in further studies.

## Supplementary Information

**Additional file 1.** Appendix A. Availability of data and materials. Appendix B. Regular expressions for toxin identication. Appendix C. Examples of actively research pathogens vs not actively researched.

## Data Availability

We have made our data sets and code available. Supplementary material file contains full listing and details for both code and data.

## Abbreviations

PMC: PubMed Central
NLP: Natural Language Processing
NRDs: Non-relevant Documents
MeSH: Medical Subject Headings
PF: Pathogen Focus
PI: Pathogen identification
PMID: PubMed Identifier (for a PubMed citation)
READBiomed: Reading, Extraction, and Annotation of Documents – Biomedical (research group)
READBiomed-Pathogens: The READBiomed group Pathogens data set produced for this research.

## Footnotes

1 https://uima.apache.org/d/uima-addons-current/ConceptMapper/ConceptMapperAnnotatorUserGuide.html

## References

[1] Balloux F, van Dorp L (2017). Q&a: what are pathogens, and what have they done to and for us?. BMC Biol.

[2] Kafkas S, Abdelhakim M, Hashish Y, Kulmanov M, Abdellatif M, Schofield PN, Hoehndorf R (2019). PathoPhenoDB, linking human pathogens to their phenotypes in support of infectious disease research. Scientific data.

[3] Neu HC (1992). The crisis in antibiotic resistance. Science.

[4] Lee SW, Kim B-S, Chen S, Shao-Horn Y, Hammond PT (2009). Layer-by-layer assembly of all carbon nanotube ultrathin films for electrochemical applications. J Am Chem Soc.

[5] Hakimi O, Krallinger M, Ginebra M-P (2020). Time to kick-start text mining for biomaterials. Nature Reviews Materials.

[6] Fata Moradali M, Rehm BHA (2020). Bacterial biopolymers: from pathogenesis to advanced materials. Nat Rev Microbiol.

[7] Wang LL, Lo K, Chandrasekhar Y, Reas R, Yang J, Burdick D, Eide D, Funk K, Katsis Y, Kinney RM, Li Y, Liu Z, Merrill W, Mooney P, Murdick DA, Rishi D, Sheehan J, Shen Z, Stilson B, et al. CORD-19: The COVID-19 Open Research Dataset. In Proceedings of the 1st Workshop on NLP for COVID-19 at ACL 2020, Online. Association for Computational Linguistics. 2020.

[8] Chen Q, Allot A, Zhiyong L (2021). LitCovid: an open database of COVID-19 literature. Nucleic Acids Res.

[9] Gardy JL, Loman NJ (2018). Towards a genomics-informed, real-time, global pathogen surveillance system. Nat Rev Genet.

[10] the NIH NCBI. READBiomed-Pathogens: https://github.com/READ-BioMed/READBiomed-Pathogens-dataset.%20Accessed%2019%20July%202022.

[11] Haas Q, Alvarez DV, Borissov N, Ferdowsi S, von Meyenn L, Trelle S, Teodoro D, Amini P (2021). Utilizing artificial intelligence to manage COVID-19 scientific evidence torrent with risklick ai: a critical tool for pharmacology and therapy development. Pharmacology.

[12] Timme RE, Balkey M, Randolph R, Venkata SLG, Wolfgang WJ, Strain EA. NCBI submission protocol for microbial pathogen surveillance v.2. Protocols io. 2020;10.

[13] Xie Y, Wei Y, Shen Y, Li X, Zhou H, Tai C, ZixinDeng, and Hong-Yu Ou. (2018). Tadb 2.0: an updated database of bacterial type II toxin–antitoxin loci. Nucleic Acids Res.

[14] Lowenthal MD, Sharples FE (2019). Developing norms for the provision of biological Laboratories in low-Resource Contexts: proceedings of a workshop.

[15] Bossy R, Deléger L, Chaix E, Ba M, Nédellec C (2019). Bacteria biotope at BioNLP open shared tasks. In: Proceedings of the 5th workshop on BioNLP open shared tasks.

[16] Molik DC, Tomlinson DA, Davitt S, Morgan EL, Sisk M, Roche B, Meyers N, Pfrender ME (2021). Combining natural language processing and metabarcoding to reveal pathogen-environment associations. PLoS Negl Trop Dis.

[17] Kafkas S, Hoehndorf R (2019). Ontology based mining of pathogen–disease associations from literature. Journal of biomedical semantics.

[18] Tahsin T, Weissenbacher D, O’Connor K, Magge A, Scotch M, Gonzalez-Hernandez G (2018). Geoboost: accelerating research involving the geospatial metadata of virus genbank records. Bioinformatics.

[19] Magge A, Weissenbacher D, O’Connor K, Tahsin T, Gonzalez-Hernandez G, Scotch M (2020). GeoBoost2: a natural language processing pipeline for GenBank metadata enrichment for virus phylogeography. Bioinformatics.

[20] Le Guillarme N, Thuiller W. TaxoNERD: deep neural models for the recognition of taxonomic entities in the ecological and evolutionary literature. Methods Ecol Evol. 2021;13(3):625–41.

[21] Chaix E, Deléger L, Bossy R, Nédellec C (2019). Text mining tools for extracting information about microbial biodiversity in food. Food Microbiol.

[22] Swaminathan S (2006). A system for discovering bioengineered threats by knowledge base driven mining of toxin data.

[23] Leaman R, Islamaj R, Lu Z (2021). The overview of the NLM-Chem BioCreative VII track full-text chemical identification and indexing in PubMed articles. In Proceedings BioCreative VII Challenge Evaluation workshop https://biocreative.bioinformatics.udel.edu/resources/publications/bc-vii-workshop-proceedings/. 10.1093/database/baad005 PMC9991492 36882099

[24] Islamaj R, Leaman R, Kim S (2021). NLM-Chem, a new resource for chemical entity recognition in PubMed full text literature. Sci Data.

[25] NCBI taxonomy: https://www.ncbi.nlm.nih.gov/taxonomy. Accessed 19 July 2022.

[26] Ecker DJ, Sampath R, Willett P, Wyatt JR, Samant V, Massire C, Hall TA, Hari K, McNeil JA, Büchen-Osmond C (2005). The microbial rosetta stone database: a compilation of global and emerging infectious microorganisms and bioterrorist threat agents. BMC Microbiol.

[27] Flanagan M, Leighton T, Dudley J. Anticipating the species jump: surveillance for emerging viral threats." Zoonoses and Public health. 2012;59(3):155–63. 10.1111/j.1863-2378.2011.01439.x PMC4948863 21914152

[28] Imran M, Mahmood S (2011). An overview of animal prison diseases. Virol J.

[29] Madsen JM, Wexler P (2005). Bio warfare and terrorism: Toxins and other mid-spectrum agents. Encyclopedia of Toxicology.

[30] Sayers E. E-utilities quick start. Entrez programming utilities help. Bethesda (MD); 2008.

[31] Charen T, CA Bachrach (1978). Selection of MEDLINE contents, the development of its thesaurus, and the indexing process. Med Inf.

[32] Benson DA, Cavanaugh M, Clark K, Karsch-Mizrachi I, Lipman DJ, Ostell J, Sayers EW (2012). GenBank. Nucleic Acids Res.

[33] Federhen S (2012). The NCBI taxonomy database. Nucleic Acids Res.

[34] Ferrucci D, Lally A, Verspoor K, Nyberg DE (2008). Unstructured information management architecture (UIMA) version 1.0.

[35] Funk C, Baumgartner W, Garcia B, Roeder C, Michael Bada K, Cohen B, Hunter LE, Verspoor K (2014). Large-scale biomedical concept recognition: an evaluation of current automatic annotators and their parameters. BMC bioinformatics.

[36] Ferrucci D, Lally A, Verspoor K, Nyberg E (2009). Unstructured information management architecture (UIMA) version 1.0.

[37] Funk C, Baumgartner W, Garcia B, Roeder C, Michael Bada K, Cohen LH, Verspoor K (2014). Large-scale biomedical concept recognition: an evaluation of current automatic annotators and their parameters. BMC Bioinformatics.

[38] Verspoor K, Roeder C, Johnson HL, Cohen KB, Baumgartner Jr WA, Hunter LE (2010). Exploring species-based strategies for gene normalization. IEEE/ACM Transactions on Computational Biology and Bioinformatics.

[39] Jimeno-Yepes A, McInnes BT, and& Alan R. (2011). Aronson exploiting MeSH indexing in MEDLINE to generate a data set for word sense disambiguation. BMC bioinformatics.

[40] Vapnik VN. The nature of statistical learning theory. New York: Springer-Verlag, Inc.; 1995.

[41] Zhang T. Solving large scale linear prediction problems using stochastic gradient descent algorithms. In: Brodley CE, editor. Machine Learning, Proceedings of the Twenty-first International Conference. ACM Press; 2004. vol. 69. 10.1145/1015330.1015332.

[42] Yeganova L, Comeau DC, Kim W, Wilbur WJ. Text mining techniques for leveraging positively labeled data. In: InProc. BioNLP 2011 Workshop. Portland: National Center for Biotechnology Information; 2011. p. 155–63.

[43] Freund Y, Schapire RE (1997). A decision-theoretic generalization of on-line learning and an application to boosting. J Comput Syst Sci.

[44] MTIMLExtension: https://github.com/READ-BioMed/MTIMLExtension. Accessed 19 July 2022.

[45] Devlin J, Chang M-W, Lee K, Toutanova K. BERT: pre-training of deep bidirectional transformers for language understanding. In: InProc. NAACL 2019: human language technologies, vol. 1. Minneapolis, Minnesota: Association for Computational Linguistics; 2019. p. 4171–86.

[46] Wolf T, Debut L, Sanh V (2020). Transformers: State-of-the-art natural language processing. In Proc. EMNLP 2020: System demonstrations.

[47] Lee J, Yoon W, Kim S, Kim D, Kim S, So CH, Kang J (2020). BioBERT: a pre-trained biomedical language representation model for biomedical text mining. Bioinformatics.

[48] Otmakhova Y, Jimeno Yepes AJ. Team ITTC at BioCreative VII LitCovid Track 5: combining pretrained and bag-of-words models. In: Proceedings of the seventh BioCreative challenge evaluation workshop. 2021.

[49] Chen Q, et al. Multi-label classification for biomedical literature: an overview of the BioCreative VII LitCovid track for COVID-19 literature topic annotations. Database. 2022;2022:baac069. 10.1093/database/baac069. 10.1093/database/baac069 PMC9428574 36043400

[50] Li X, Burns G, Peng N. Scientific Discourse Tagging for Evidence Extraction. In Proceedings of the 16th Conference of the European Chapter of the Association for Computational Linguistics: Main Volume, Online. Association for Computational Linguistics; 2021. p. 2550–62.

[51] Yepes AJ, Albahem A, Verspoor K (2021). Using discourse structure to differentiate focus entities from background entities in scientific literature. In Proc. (ALTA 2021).

[52] Jacquemin C (1994). FASTR: a unification-based front-end to automatic indexing. Proc.

[53] Verspoor K, Šuster S, Otmakhova Y, Mendis S, Zhai Z, Fang B, Lau JH, Baldwin T, Yepes AJ, Martinez D (2021). Brief description of COVID-SEE: the scientific evidence explorer for COVID-19 related research. European conference on information retrieval.

[54] Tanenblatt MA, Coden A, Sominsky IL. The ConceptMapper approach to named entity recognition. In: Proceedings of the Seventh International Conference on Language Resources and Evaluation (LREC'10), Valletta, Malta. European Language Resources Association (ELRA).

