# Supplementary material for: Classifying literature mentions of biological pathogens as experimentally studied using natural language processing
Source: J Biomed Semantics. 2023 Jan 31;14:1. doi: 10.1186/s13326-023-00282-y (PMC9889128; doi:10.1186/s13326-023-00282-y)
Supplement: Supplementary file 1 — Additional file 1. Appendix A. Availability of data and materials. Appendix B. Regular expressions for toxin identication. Appendix C. Examples of actively research pathogens vs not actively researched. [file 13326_2023_282_MOESM1_ESM.pdf]

Supplementary material - Classifying literature mentions of biological pathogens as experimentally studied using natural language processing

Appendix A. Availability of data and materials

The code has been made publicly available from a GitHub repository.

- Code developed in the project to build the pathogen characterisation data set is available from: `https://github.com/READ-BioMed/readbiomed-ncbi-pathogen-dataset-generation`
- Code for the pathogen characterisation methods is available at: `https://github.com/READ-BioMed/readbiomed-pathogen-annotator`.
- The MTIMLExtension package used to train text classifiers with SVM and AdaBoostM1 are available from: `https://github.com/READ-BioMed/MTIMLExtension`.

In addition to code, we have made available from GitHub data used in this project, which includes the data sets that we have created using NCBI resources.

- The data sets created for this project have been made available from: `https://github.com/READ-BioMed/readbiomed-pathogens-dataset`.

Appendix B. Regular expressions for toxin identification

The regular expressions below have been tested with the Java programming language for the identification of toxins in MEDLINE citations.

(?i)(Abrus.*abrin.*toxin)
(?i)(Aflatoxin)
(?i)(Anatoxin-A)
(?i)(Batrachotoxin)
(?i)(Botulinum.*toxin)

(?i)(Brevetoxin)
(?i)(Ciguatoxin)
(?i)(Conotoxin)
(?i)(decarbamoylsaxitoxin)
(?i)(Fusariotoxins.*(T-2))
(?i)(gonyautoxin)
(?i)(Maitotoxin)
(?i)(Mycotoxin)
(?i)(neosaxitoxin)
(?i)(Palytoxin)
(?i)(Ricinus.*ricin.*toxin)
(?i)(saxitoxin)
(?i)(Staphylococcus.*enterotoxin—Enterotoxin)
(?i)(Tetrodotoxin)

Appendix C. Examples of actively research pathogens vs not actively researched

We show information about five MEDLINE citations to highlight the difference between pathogen mentions that discuss viruses actively researched in the study discussed in a citation and pathogens mentioned in a citation that is not the actively studied pathogen.

The first one (PMID: 19040284) discusses work about the *hepatitis delta virus*. Samples of the virus were collected and analysed.

The second one (PMID: 27756212) mentions the *Japanese encephalitis virus (JEV)* in the context of studying its vaccine for the dengue virus. In this paper, JEV is referenced in the citation but it is not the pathogen of study in the paper.

The third one (PMID: 7033132) mentions the toxin *C2* that is produced by *Clostridium botulinum types C and D*. In this research, the toxin and the bacteria are relevant and actively studied.

The fourth one (PMID: 31694513) discusses changes in the receptor-binding properties of H3N2 viruses. In this case, the H3N2 virus will be considered as an actively researched pathogen.

The fifth one (PMID: 21979562) mentions the H1N1 virus, but the virus is mentioned in the context of the clinical presentation in which the patient had a vaccination against the influenza A H1N1 virus.

PMID: 19040284
Title: Molecular epidemiology of **hepatitis delta virus (HDV)** in Iran: a preliminary report
Abstract: To identify hepatitis delta virus (HDV) genetic variability and its circulating genotypes amongst infected Iranian patients, 25 patients with positive anti-HDV status from different parts of Iran were enrolled in this cross-sectional study. A portion of the HDV delta antigen was amplified, sequenced, and subjected to molecular and phylogenetic analysis. Clinical features and virological markers were evaluated. HDV RNA could be detected in 88% of anti-HDV positive cases (22 patients) with chronic hepatitis B virus (HBV) infection and liver cirrhosis. Phylogenetic analysis revealed that all Iranian patients were infected by genotype I (clade 1) of HDV, supported by a high bootstrap value (100%, 1,000 replicates). All HDV-positive patients were coinfected with genotype D1 of HBV. No significant association was determined between demographic, clinical, and virological variables in the population studied. In conclusion, the present molecular epidemiology survey reveals that clade 1 of HDV is predominant among coinfected HBV patients in Iran.

PMID: 27756212
Title: Japanese encephalitis vaccine-facilitated dengue virus infection-enhancement antibody in adults
Abstract:
Background: Dengue virus (DENV) and Japanese encephalitis virus (JEV) belong to the genus Flavivirus, and infection with a virus within this genus induces antibodies that are cross-reactive to other flaviviruses. Particularly in DENV infection, antibodies to DENV possess two competing activities: neutralizing activity and infection-enhancing activity. These antibody activities are considered central in modulating clinical outcomes of DENV infection. Here, we determined the neutralizing and infection-enhancing activity of DENV cross-reactive antibodies in adults before and after JE vaccination.
Methods: Participants were 77 Japanese adults who had received a single dose of inactivated Vero cell-derived JE vaccine. A total of 154 serum samples were obtained either before or approximately a month after a single dose of JE vaccination. The antibody-dependent enhancement (ADE) activity to each of four DENV serotypes and the neutralizing activities to DENV and to JEV were determined in each of the serum samples by using baby hamster kidney (BHK) cells and FcγR-expressing BHK cells.
Results: A total of 18 post-JE immunization samples demonstrated cross-reactivity to DENV in an anti-DENV IgG ELISA. DENV neutralizing antibodies were not detected after JE vaccination in this study. However, undiluted post-JE vaccination serum samples from 26 participants demonstrated monotypic and heterotypic ADE activity to DENV. ADE activity was also observed in 1:10-diluted samples from 35 of the JE vaccine recipients (35/77, 45%).
Conclusion: In summary, JE vaccination induced DENV cross-reactive antibodies, and at sub-neutralizing levels, these DENV cross-reactive antibodies possess DENV infection-enhancement activity. The results also indicate that cross-reactivity to DENV is associated with high levels of JEV neutralizing antibodies and, the DENV cross-reactivity is further facilitated by JE vaccination.

PMID: 7033132
Title: Production of C2 toxin by Clostridium botulinum types C and D as determined by its vascular permeability activity
Abstract:
Vascular permeability (VP) activity was demonstrated by intradermal injection of culture supernatants of Clostridium botulinum types C and D and strains producing only C2 toxin. The activity was enhanced markedly by treatment with trypsin. It was abolished by antiserum against C2 toxin and by antisera specific for components I and II of C2 toxin, but not by anti-type C or -type D neurotoxin serum. Of 14 strains examined, 10 had VP activity. No VP activity was demonstrated in the culture supernatants of C. botulinum type A, B, E, or F. These results indicate that VP activity is a function of the C2 toxin elaborated by C. botulinum types C and D and that the toxin possesses VP as well as lethal activities. These findings raise the possibility that VP activity of C2 toxin exerts synergic effect(s) with neurotoxin in the pathogenesis of botulism caused by type C and D strains.

PMID: 31694513
Title: Changes in the Receptor-Binding Properties of H3N2 Viruses during Long-Term Circulation in Humans
Abstract:
It was previously shown that hemagglutinin residues Thr155, Glu158, and Ser228 are crucial for the recognition of Neu5Gc. In this study, we demonstrated that the ability to bind the Neu5Gc-terminated receptor is related to the amino acid 145: viruses of years 1972-1999 with Lys145 bind to the receptor, whereas viruses with Asn145 do not. Sporadic appearance and disappearance of the ability to bind Neu5Gc oligosaccharides and the absence of Neu5Gc in the composition of human glycoconjugates indicate the non-adaptive nature of this ability. It was previously shown that unlike H1N1 viruses, H3N2 viruses of years 1968-1989 did not distinguish between Neu5Ac2-6Gal1-4Glc (6'SL) and Neu5Ac2-6Gal1-4GlcNAc (6'SLN). H3N2 viruses isolated after 1993 have acquired the ability to distinguish between 6'SL and 6'SLN, similarly to H1N1 viruses. We found that the affinity for 6'SLN has gradually increased from 1992 to 2003. After 2003, the viruses lost the ability to bind a number of sialosides, including 6'SL, that were good receptors for earlier H3N2 viruses, and retained high affinity for 6'SLN only, which correlated with the acquisition of new glycosylation sites at positions 122, 133, and 144, as well as Glu190Asp and Gly225Asp substitutions, in hemagglutinin. These substitutions are also responsible for the receptor-binding phenotype of human H1N1 viruses. We conclude that the convergent evolution of the receptor specificity of the H1N1 and H3N2 viruses indicates that 6'SLN is the optimal natural human receptor for influenza viruses.

PMID: 21979562

Title: A novel mutation and unusual clinical features in a patient with immune dysregulation, polyendocrinopathy, enteropathy, X-linked (IPEX) syndrome

Abstract:

We report a patient with immune dysregulation, polyendocrinopathy, enteropathy, X-linked (IPEX) syndrome with a novel splicing mutation of the FOXP3 gene. The patient is a boy, born at 39 + 2 weeks gestation with a birth weight of 3,280 g. The family history was unremarkable. He was well until 11 months of age, when he was diagnosed with type 1 diabetes mellitus. The level of urine C-peptide was 0.58 g/day (normal range, 44-116 g/day). Glutamic acid decarboxylase autoantibody was not detected, but a high level of anti-insulin antibody (50 IU/mL; normal range, ¡5 IU/mL) was noted. This patient presented with unusual clinical features, including pure red cell aplasia, membranous glomerulopathy, and posterior reversible encephalopathy syndrome after a vaccination against influenza A H1N1 virus. The diagnosis of IPEX was made when the patient was 11 years old, which is quite late compared with typical cases.

Conclusion: Although IPEX syndrome is usually a disease of infancy, it should not be ruled out solely on the basis of age. IPEX presentation is so variable that it should be suspected in a male child with one or more autoimmune disorders and severe infections.
